# Activity of PROTAC MDM2 degrader in primary leukemia cells and PDX models

**DOI:** 10.1038/s41375-026-02957-8

**Published:** 2026-04-15

**Authors:** Malathi Kandarpa, Luke F. Peterson, Harish Potu, Megha Ramappan, Yihong Liu, Avery Polk, Shaomeng Wang, Moshe Talpaz

**Affiliations:** 1 Department of Internal Medicine, Division of Hematology & Oncology, University of Michigan Medical School, Ann Arbor, MI USA; 2 Rogel Cancer Center, The University of Michigan - Michigan Medicine, Ann Arbor, MI USA; 3 Department of Pharmacology, Department of Medicinal Chemistry, University of Michigan Medical School, Ann Arbor, MI USA

**Keywords:** Targeted therapies, Leukaemia

## Abstract

MDM2 is an E3 ubiquitin ligase that promotes p53 tumor suppressor degradation and has emerged as a therapeutic target in the treatment of wild-type (wt) TP53 tumors. In acute myeloid leukemia (AML), TP53 mutations are infrequent (15–20%), but wt-p53 is often inactivated through overexpression of MDM2. Thus, MDM2 inhibitors are currently in clinical trials for AML. However, p53 stabilization with inhibitors upregulates MDM2, which limits their clinical efficacy. Proteolysis-targeting chimeric (PROTAC) molecules that degrade MDM2 may overcome this feedback. MD-265 is a PROTAC that recruits CRBN, degrades MDM2, restores p53 and induces apoptosis. We tested MD-265 in ex vivo cultures of 105 primary leukemic stem cells (LSCs). The median cytotoxic IC_50_ for MD-265 was 16 nM, median IC_50_ for MI-1061 was 150-fold higher. LSCs with IC_50_ > 1 µM were classified as MD-265 resistant and harbored mutations in TP53. Normal hematopoietic stem cells showed 100-fold higher IC_50_ (818 nM) than LSCs. AML patient-derived xenograft (PDX) models in NSG-SGM3 mice were treated with MD-265 or an oral MDM2 inhibitor. In PDX models, MD-265 was not toxic and prolonged survival. MD-265 is a potent and specific MDM2 degrader with broad pre-clinical activity and a promising drug candidate for the treatment of leukemias.

## Introduction

Acute myeloid leukemia (AML) remains a leading hematologic malignancy with poor outcomes for many patients despite incremental gains from anthracycline-based regimens [1, 2]. Genomic profiling has prompted mechanism-directed therapies for subsets of AML, including inhibitors targeting FLT3 [3], IDH1/2 [4], menin-KMT2A (MLL1) interactions [5], epigenetic modulators, and apoptosis pathways such as Bcl2 and Mcl1 [6]. Nevertheless, benefits are often confined to molecularly defined subgroups, underscoring the need for approaches with broader applicability.

TP53 is a critical tumor suppressor gene that is commonly inactivated in leukemia cells by mutation or deletion, leading to tumor progression. The p53 protein coordinates cell cycle progression (p21), DNA damage responses (Ku70), apoptosis (BAX, PUMA), and senescence (PAI-1) [7]. In AML, TP53 alterations occur less frequently than in many solid tumors, with rates ranging from 5% in de novo disease to 30% in therapy-related cases [8]. More commonly, the E3 ubiquitin ligase MDM2 is overexpressed in ~50% of leukemias and promotes ubiquitination and proteasomal degradation of p53 [9, 10]. Small-molecule MDM2 inhibitors that disrupt the MDM2-p53 interaction have [10] shown clinical activity in leukemias [11, 12]. However, p53 stabilization transcriptionally upregulates MDM2, which can blunt responses and foster resistance [13]. Moreover, overexpression of MDM2 often co-occurs with unfavorable cytogenetic features in leukemias. To augment the activity of MDM2 inhibitors, we and others are studying more potent compounds that degrade MDM2 and prevent the feedback increase in MDM2 levels.

Targeted protein degradation offers an alternative means to suppress disease drivers. Proteolysis-targeting chimeras (PROTACs) are heterobifunctional molecules that bind a target protein and an E3 ligase, inducing ubiquitination and proteasomal degradation of the target [14]. PROTACs targeting oncogenic proteins such as BCR::ABL1 are in development for the treatment of chronic myeloid leukemia [15]. MD-265 was recently developed as a PROTAC degrader of MDM2, where the MDM2 inhibitor (MI-1061) is linked with lenalidomide [16]. Lenalidomide recruits the Cullin–RING ligase 4–cereblon (CRL4–CRBN) ligase complex to MDM2, leading to the degradation of MDM2. MD-265 depletes MDM2 protein in cancer cells at 1-2 nM, leading to activation of p53 in cells carrying wt-p53 [16]. It selectively inhibited the growth of wt-p53 leukemic cell lines with no effect on mutated p53 cell lines. MD-265 demonstrated tumor regression with acceptable tolerability in a cell-line leukemia xenograft model, markedly prolonging the overall survival of mice [16].

Here we evaluate the pre-clinical efficacy of MD-265 in a large, molecularly diverse cohort of primary AML leukemic stem cells (LSCs). We show that MD-265 potently degrades MDM2, robustly induces p53, and triggers apoptosis ex vivo across AML subtypes. Among the few resistant AMLs, p53 mutation and lack of MDM2 expression likely play a role. MD-265 had reduced activity in normal hematopoietic stem and progenitor cells (HSPCs), suggesting a therapeutic index. Finally, we demonstrate in vivo activity of MD-265 in patient-derived xenograft models of AML.

## Materials, subjects and methods

### Patient samples, characteristics and clinical data

Patient samples were acquired from Michigan Medicine, Ann Arbor, MI, under protocols approved by the University of Michigan Institutional Review Board. All patients provided written informed consent for banking and molecular analyses in accordance with the Declaration of Helsinki. Clinical data, including blood counts, previous treatment history, cytogenetics and mutational data, were abstracted from patient charts.

### Isolation and growth of primary AML stem cells and normal stem cells

Peripheral blood or bone marrow aspirates were collected from leukemia patients and processed by Ficoll density-gradient centrifugation to isolate mononuclear cells. Leukemic stem cells (LSCs) were enriched by magnetic-activated cell sorting (MACS, Miltenyi Biotec) for CD34-positive cells. Enrichment (70% to 98%) was confirmed by flow cytometry with anti-CD34-PE antibody (Biolegend). Normal CD34-positive cells were obtained from mobilized peripheral blood or umbilical cord blood from healthy donors. Normal or leukemic stem cells were plated in Iscove’s medium containing granulocyte macrophage colony stimulating factor (GMCSF) 200 pg/mL, granulocyte colony stimulating factor (GCSF) 1 ng/mL, stem cell factor (SCF) 200 pg/mL, leukemia inhibitory factor (LIF) 50 pg/mL, macrophage inflammatory protein a (MIP1α) 200 pg/mL, interleukin 6 (IL-6) 1 ng/mL and 5% BIT (Stem Cell Technology) [17].

### Dose-response assays and apoptosis

LSCs were exposed to varying concentrations of MDM2 inhibitor or degrader and incubated for 72 h in cytokine-containing media as described above. Cell death and apoptosis were detected by Annexin V-FITC (Biolegend) and DAPI staining, followed by flow cytometry. Percentages of live cells were normalized to untreated controls. Cytotoxicity and survival curves were fitted to derive IC50 values.

### Colony-forming cell (CFC) assays

CD34+ cells from AML patients or normal donors (30,000-50,000 cells/well) were suspended in human methylcellulose complete media (R&D Systems) containing the indicated drugs and plated in 12-well plates in duplicate. Colonies were scored after 2 weeks of incubation in a CO_2_ incubator.

### TP53 sequencing and expression of MDM2 and MDM4

DNA was prepared from degrader-resistant AML blasts by proteinase K digestion. TP53 exons 3-9 were amplified by PCR primers described previously [18], and Sanger sequenced. Alignments to wt TP53 and variant cells were performed using SeqMan Pro (DNASTAR) software.

For MDM2 and MDM4 gene expression analysis, RNA was isolated from AML blasts of MDM2 degrader-resistant samples. After reverse transcription, real-time quantitative PCR (Real Time Primers) was performed to measure MDM2 and MDM4 transcripts in the samples.

### Immunoblotting for inhibitor and degrader targets

LSCs were cultured with cytokines (G-CSF, GM-CSF, MIP-1a, SCF, LIF, IL-6) and treated for 4 h with the indicated drug concentrations. Cell lysates were separated by SDS-PAGE and transferred for immunoblotting with antibodies against p53 (clone DO-1, Sigma-Aldrich), MDM2 (clone 4B11, Sigma-Aldrich), MDM4 (clone 7A8, Sigma-Aldrich) and β-actin (AC-15; Sigma-Aldrich).

### PDX models of AML

All mouse experiments were approved by the institutional animal care and use committee. Cryopreserved CD34-positive-enriched AML cells were thawed the day before transplant. NSG-SGM3 mice (Jackson Laboratory), 6 to 8 weeks old, received sublethal irradiation (90 cGy) on the day of transplant, followed by tail-vein injection of 500,000 to 1 million cells. Engraftment was monitored every 3 weeks by flow cytometry of peripheral blood using anti-mouse CD45-FITC (Biolegend) and anti-human CD45-PE (Clone 2D1, Biolegend). Upon detection of 2–10% human CD45+ cells, mice were randomized to vehicle, MD-265 (25 mg/kg by IV once a week), or oral APG-115 (200 mg/kg 3 times a week). Blood was drawn via the tail vein every week during treatment to quantify human leukemic cells by flow cytometry.

### Statistical methods

Dose-response curves were fitted in GraphPad Prism using nonlinear regression with Hill slope. Significant differences in cell survival between sensitive and resistant AML LSCs, and between AML LSCs and normal HSPCs were calculated using the log-rank (Mantel-Cox) test; *P* values of less than 0.05 were considered statistically significant. Mouse survival data were analyzed by the log-rank (Mantel-Cox) test in GraphPad Prism.

## Results

### Patient cohort characteristics

Response to AML therapy often varies among patients even under equivalent therapeutic regimens. Molecular and genetic disease features, as well as patient characteristics, contribute to this heterogeneity [19]. MD-265 is an MDM2 degrader in pre-clinical development for AML [16]. To advance this class of therapeutic agents, we evaluated the pre-clinical ex vivo efficacy of MDM2 degrader in 108 primary, patient-derived leukemic stem and progenitor cell samples. We focused primarily on AML (*N* = 100): 84 were newly diagnosed, and 16 had relapsed or were refractory to treatment at the time of sample collection. Secondary disease status refers to patients who had an AML diagnosis secondary to Myelodysplastic syndrome, myeloproliferative disease or therapy-related AML after treatment for another malignancy such as myeloma, lymphoma, breast cancer or prostate cancer. The demographics and clinical characteristics of the patients are summarized in Table 1, and reflect a higher number of males (62%) and intermediate cytogenetic risk patients (*N* = 58), with most having a normal karyotype [20].

**Table 1:** Patient Cohort Characteristics.

Cohort Characteristics		*N* = 108
**Disease**	AML	100
	B-ALL	5
	APML	1
	CML	1
	MDS/MPN	1
**Age, median years (IQR)**		66 [23]
**Male gender (%)**		62%
**Disease Status at sample collection**	New Diagnosis	84
	Relapse/Refractory	16
	Secondary	26
**Cytogenetics,** *n*	Favorable	13
	Intermediate	58
	Adverse	27
	Unknown	10
**WBC (x 10**^**9**^ **/L)**	>40 × 10^9^/L	40
	>100 × 10^9^/L	10
**Early mortality,** *n*	30-day mortality	18
	90-day mortality	30
**TP53 inactivation**		11

### MDM2 degrader is more effective in primary LSCs than normal HSPCs

**Fig. 1:**
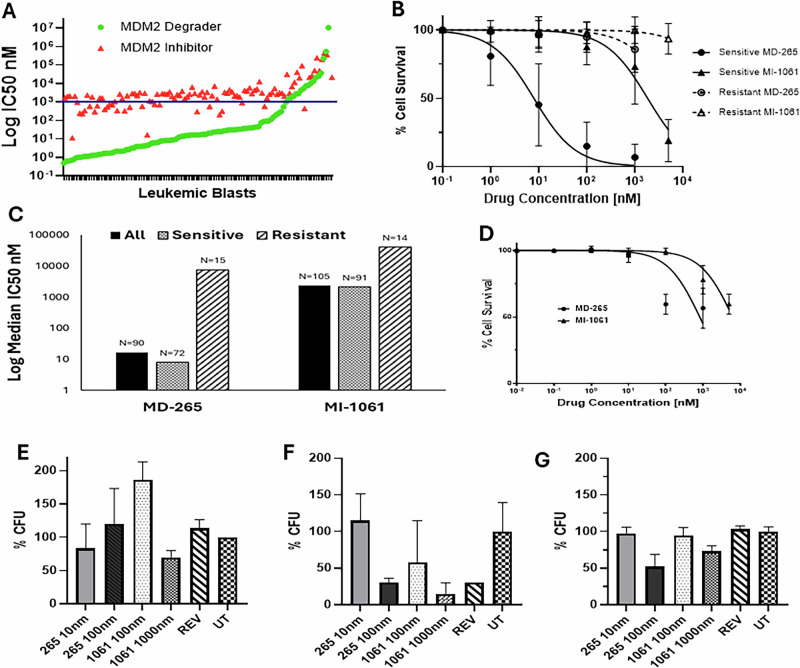
Efficacy of MDM2 degrader and inhibitor in 100 primary AML stem cells. **A** IC50 values for MDM2 degrader MD-265 (green circles) and MDM2 inhibitor MI-1061 (red triangles) in primary leukemic stem cells (*N* = 100). The values above the line are samples with relative resistance to the inhibitor and degrader. Patient samples resistant to the degrader were also resistant to the inhibitor. **B** Dose-response curves for MD-265 and MI-1061 in primary LSCs; green curves denote relatively resistant samples. **C** Median IC50 for MD-265 (MDM2 degrader) and MI-1061 (MDM2 inhibitor) in patient leukemic blasts. The sample size is indicated on top of the bars. Among resistant blasts, the LSCs that had no measurable response were excluded as their predicted IC50 was infinite. **D** Dose-response curves and IC50 (nM) for MD-265 (circles) and MI-1061 (triangles) in normal HSPCs. **E** Colony-forming unit (CFU) assay in methylcellulose for one representative sensitive AML (AML-60). Treatments are indicated under the bars: MD265 at 10 nM and 100 nM, MI1061 at 100 nM and 1000 nM, Revlimid alone at 20 nM and UT (no drug control). **F** CFU for a resistant AML (AML-491). **G** Colony-forming units from CD34+ HSPCs from a normal donor (N1376). For (**E**, **F**, **G**) cells were plated in duplicate wells, treatments are indicated under the bar, and averages are shown with standard deviations. For (**G**), one of three different normal CD34-positive HSPCs tested is shown.

PROTAC-based MDM2 degraders LE-004 and MD-265 were designed based on the MDM2 inhibitor MI-1061 [9, 10]. MD-265 demonstrated improved pharmacologic properties over LE-004 and was therefore the focus of these studies. These drugs had comparable in vitro efficacy. To evaluate the effect of MDM2 degraders on LSCs, peripheral blood cells obtained from AML patients were enriched by MACS section of CD34-positive cells, or samples with >80% CD33+ or CD34+ blasts were used and cultured in serum-free media with cytokines (G-CSF, GM-CSF, MIP-1a, SCF, LIF, and IL-6). Survival dose-response to MD-265 (*N* = 93), LE-004 (*N* = 17) and the MDM2 inhibitor MI-1061 (*N* = 110) was determined (Fig. 1A, B and Supplementary Fig. 1). The median cytotoxic IC_50_ for MD-265 was 16 nM; in contrast, the median IC_50_ for MI-1061 was 150-fold higher at 2.3 μM. AML LSCs were classified as sensitive and resistant to MD-265 using a threshold IC_50_ of ≤1 μM (sensitive) vs >1 uM (resistant). The median IC_50_ of sensitive LSCs was 1000-fold lower than that of resistant LSCs. LSCs sensitive to the MDM2 degrader were also sensitive to the inhibitor.

Survival and dose response of MD-265 in normal CD34+ HSPCs were determined (Fig. 1D). Under similar conditions, normal CD34 cells were 100-fold less sensitive to MD-265, compared to sensitive AML blasts. CFU formation from CD34+ cells was inhibited by MD-265 treatment in a dose-dependent fashion (Fig. 1G).

### Mechanisms of resistance to MDM2 inhibition/degradation in leukemia include mutant p53 and low MDM2/MDM4 expression

Table 2 shows sequencing results for TP53 exons 3-9 in primary leukemic samples resistant to the inhibitor and degrader. In addition to activating mutations, LSCs from two patients harbored TP53 deletions, and six samples showed very low MDM2 and MDM4 expression by real-time quantitative PCR and undetectable protein by immunoblotting (Supplementary Fig. 2).

**Table 2:** TP53 mutational analysis and gene expression of MDM2 and MDM4 in leukemic blasts resistant to MDM2 inhibitor and degrader.

Sample ID	TP53 status	Expression of MDM2 and MDM4
**AML-689**	Exon 5 Y220C	Low
**AML-441**	Deletion	High
**AML-926**	Exon 7 G245A	
**AML-567**	Exon 7 D259V	
**AML-287**	None detected	Low
**AML-220**	Exon 3 F113C	
**AML-194**^a^	Deletion	Low
**AML-659**	Exon 7 D250L	
**AML-478**	Exon 5 P151S	
**AML-78**	Exon 1 R65I, Exon 7 R248W	
**AML-255**	Exon 7 R248G, Exon 8 R273C	
**AML-754**	Exon 8 D281G	
**AML-217**	None detected	Low
**AML-913**	None detected	Low
**AML-703**	None detected	Low

^a^Low sensitivity to degrader (IC50 46 nM) and resistant to inhibitor

### MDM2 inhibition/degradation is effective in a broad range of leukemias, irrespective of molecular subtype

**Fig. 2:**
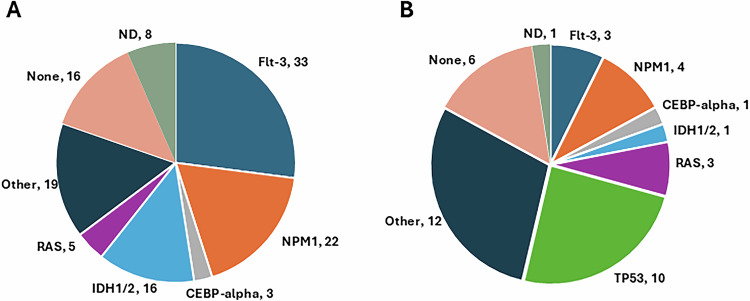
Molecular features of primary leukemia samples stratified by response to MDM2 targeting. **A** Molecular characteristics and their frequencies in samples sensitive to the MDM2 degrader. The number in the label depicts the number of samples that carried the gene variant. None and not determined (ND) do not exclude additional mutations. Other mutations include GATA2, SETD2, EPHA2, PMS2, GEN1, JAK2, RUNX1, PRPF40b, SRSF2, ERB4, **B** Molecular characteristics and their frequency in resistant samples. Other mutations include GATA2, SRSF2, NSD1, JAK2, DPYD, NTRK1.

AML is a heterogeneous disease with many phenotypic and molecular cohorts. We sought to identify markers that correlate with sensitivity or resistance to MDM2-targeting agents. We focused on Flt3, NPM1, CEBP-alpha, IDH1/2 as the most commonly mutated driver genes in AML [2]. Molecular characteristics of primary leukemia samples sensitive to the MDM2 inhibitor and degrader are shown in Fig. 2A. All subsets were comparably responsive to both agents, as shown in Fig. 2B. All mutational subtypes were represented among resistant samples. Of note is Ras mutant AML with poor prognostic implications and hard to treat even with highly active targeted agents such as venetoclax [21]. Ras mutations were found in both degrader-sensitive and resistant AML cohorts. As described earlier, mutant TP53 and deletion of TP53 are known to be resistant to MDM2 inhibitors and degraders. The degrader-resistant cohort included TP53 variants that were absent in the sensitive cohort. Other gene variants observed in the sensitive cohort included JAK2, SRSF2, SETD2, EPHA2, KMT2A fusions, and CBFB-MYH11, some of which were also found in the resistant cohort. Small sample size precludes any significant association of the MDM2 degrader resistance with genetic variants other than TP53.

### Unlike MDM2 inhibitor, the MDM2 degrader does not increase MDM2 levels

**Fig. 3:**
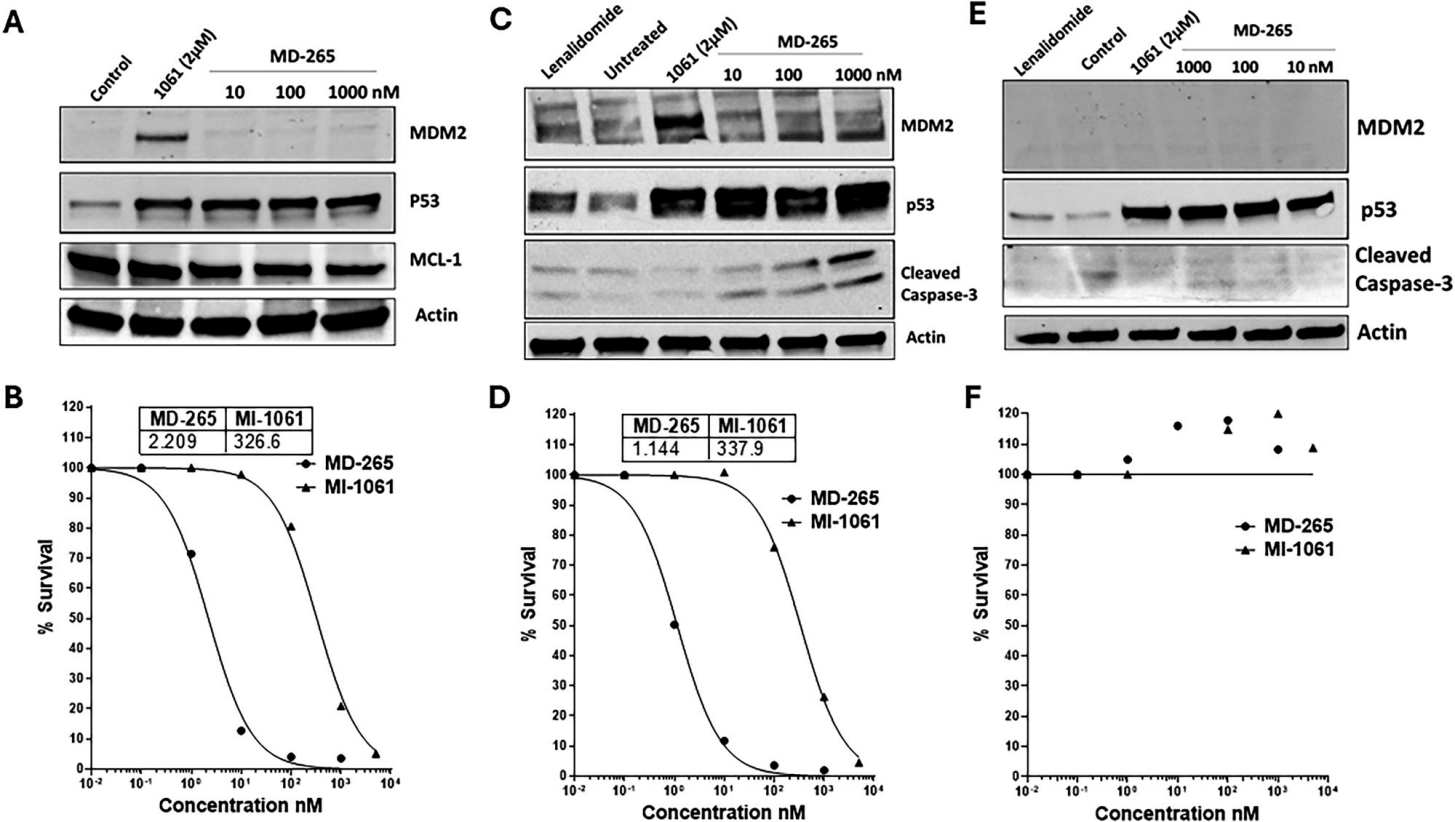
MDM2 degrader depletes MDM2 protein and induces apoptosis in AML patient-derived LSCs. **A** Immunoblots of sensitive LSCs (AML-807) treated for 4 h with MD-265 or MI-1061 for MDM2 and p53; MCL-1, as a marker of apoptosis, is also shown; β-actin was used as a loading control. **B** Dose-response curves for MD-265 (circles) and MI-1061 (triangles) in LSCs (AML-807). **C** Immunoblots of sensitive primary LSCs (AML-397) treated for 4 h with MD-265 or MI-1061. MDM2, p53 and cleaved caspase-3 are shown as an indicator of apoptosis. **D** Dose-response curves for MD-265 (circles) and MI-1061 (triangles) in LSCs (AML-397). **E** Immunoblots to resistant primary AML (AML-703), MDM2 protein, p53 levels and Caspase-3 cleavage in inhibitor and degrader-treated cells (4 h) are shown. **F** Dose-response in resistant LSCs (AML-703) to MD-265 (circles) and MI-1061 (triangles). In (**B**, **D**, **F**) The dose response was measured after treatment for 72 h. Calculated IC50 values are shown in inset table.

As a result of p53 stabilization by MDM2 inhibition, MDM2 expression is upregulated by a feedback loop mechanism [22]. This upregulation can limit the clinical efficacy of the inhibitors and promote resistance. In contrast, primary AML blasts treated ex vivo with MD-265 did not show increases in MDM2 levels (Fig. 3A, B). Other MDM2 degraders also showed a lack of MDM2 overexpression in cell line models [16, 23]. MDM2 gene expression significantly increased with inhibitor treatment but not with degrader (Supplementary Fig. 3). A robust p53 induction in sensitive LSCs was observed (Fig. 3A, B). Degrader-resistant AML blasts, that did not have a mutation in TP53, had undetectable levels of MDM2 expression as determined by immunoblots (Fig. 3C) (AML-703, in Table 2). These resistant blasts had low gene expression of MDM2 and MDM4 (Supplementary Fig. 2B). Although they show some increase in p53 induction after treatment, this did not result in any cell death as determined by lack of caspase-3 cleavage. Sensitive AML blasts showed reduced Mcl-1 [24], an anti-apoptotic protein (Fig. 3A). It is well established that p53-mediated apoptosis induction leads to caspase-3 cleavage [25], similar caspase-3 cleavage increase was observed in AML blasts treated with MDM2 degrader MD-265 (Fig. 3B). Cytotoxic dose response measured by Annexin V and DAPI staining showed dose-dependent decrease in cell survival in sensitive AML blasts (Fig. 3D, E). There was no cell killing with the degrader and inhibitor in resistant blasts, even at high doses of drug (Fig. 3F).

### MDM2 degrader MD-265 confers a significant survival benefit in patient-derived xenograft (PDX) models

**Fig. 4:**
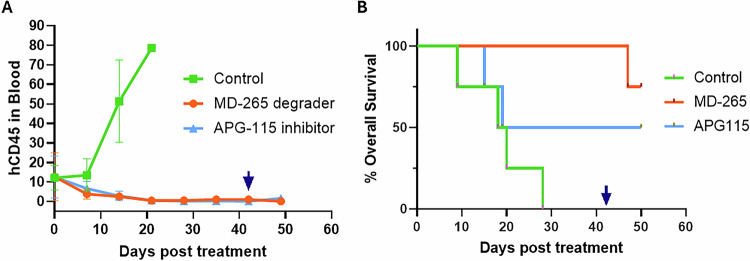
Efficacy of MD-265 in the PDX model of AML. LSCs (CD34 positive) derived from an AML patient (AML-587) were injected into NSG-S mice via the tail vein, and treatment was started after the establishment of human cells detectable in the blood. After disease development, 4 mice were assigned to each treatment group: Vehicle, MD-265 and APG-115. **A** Human CD45-positive cells were measured in peripheral blood by flow cytometry weekly. Dosing: MD-265 25 mg/kg IV once a week; MDM2i, APG115, 200 mg/kg PO three times weekly. **B** Kaplan–Meier survival of mice bearing AML PDX. Median survival for vehicle-treated mice was 19 days, for APG-115 treated 345 days and for MD265 was undefined. Based on the Log-rank (Mantel-Cox) test, the survival difference *p* value was 0.0475. The arrow in A and B indicates when treatment was discontinued. Early mortality in mice treated with MDM2 inhibitor MI-1061 was associated with perforation during gavage and rapid loss of weight. AML-587 was associated with NPM1 and FLT3-ITD mutation with normal cytogenetics. One of 3 PDXs is shown in the figures.

We next tested MD-265 in mice bearing human AML PDX models established in NSG-SGM3 mice. When the tumor burden reached 10% in the mouse peripheral blood, mice received MD-265 by IV once a week or APG115 by oral gavage 3 times a week. APG115 is a derivative of the inhibitor MI-1061, synthesized after extensive SAR and has very high affinity to MDM2 (K_i_<1 nM), potent cellular activity (60 nM IC50), an excellent oral pharmacokinetic profile, and shows tumor regression in mouse xenograft models [26]. APG-115 is currently in clinical trials; therefore, we chose this inhibitor for comparison in PDX models. Both treatments reduced xenograft tumor growth rate, measured by the frequency of human CD45-expressing cells in mouse blood (Fig. 4A). A complete reduction in tumor burden (below the detection limit by flow cytometry) was observed within 3 weeks of treatment. After 7 weeks, treatment was stopped. Inhibitor-treated mice were rapidly repopulated with human leukemia cells, whereas degrader-treated mice exhibited slower expansion. MD-265 prolonged overall survival compared with both vehicle and inhibitor (Fig. 4B). Longer-term APG115 treatment was associated with weight loss, whereas MD-265 treatment did not show any weight loss (Supplementary Fig. 4A, B).

### Ethics approval and consent to participate

All methods were performed in accordance with the relevant guidelines and regulations. Approval has been obtained from the University of Michigan Institutional Review Board for enrollment in a repository study (HUM00066564) and the use of human samples for research (HUM00202519). All animal studies were performed with approval from the Institutional Animal Care & Use Committee of the University of Michigan (PRO00011869). Patient samples were collected after informed consent was obtained from all participants in the repository study.

## Discussion

MDM family proteins consisting of MDM2 and MDM4 (MDMX) regulate the tumor suppressor p53 [27]. In many cancers, MDM2 overexpression inactivates p53, promoting tumor growth. MDM2 degraders are designed to eliminate MDM2 protein, allowing p53 to exert its tumor-suppressive functions [28], including cell cycle arrest, DNA repair, and apoptosis. These targeted degraders bring MDM2 to an E3 ubiquitin ligase, marking it for proteasomal degradation [29]. In our cohort, the degrader MD-265 demonstrated nanomolar potency against primary human leukemia cells (Fig. 1), whereas the MDM2 inhibitor exhibited micromolar activity (Fig. 1) [30]. The higher potency of MDM2 degraders may offer therapeutic advantages, such as improved efficacy at lower doses with fewer adverse effects. Unlike MDM2 inhibitors, which can induce compensatory upregulation of MDM2, MDM2 degraders reduce MDM2 levels and produce greater p53 induction, potentially enhancing antitumor activity.

The differential sensitivity of normal HSPCs vs LSCs to an MDM2 degrader suggests a favorable therapeutic index. In our ex vivo assays, normal CD34+ HSPCs were 5-fold less sensitive to MD-265 than AML LSCs (Fig. 1D, F), indicating that antileukemic responses may be achievable at doses with limited suppression of normal hematopoiesis. This selectivity is clinically meaningful as it should reduce the likelihood of systemic side effects, such as marrow toxicity, thereby improving the feasibility of sustained dosing and combination strategies.

MDM2 inhibition/degradation demonstrated activity across a broad range of molecular subtypes (Fig. 2). In high-risk cohorts such as FLT3-ITD mutant and Ras mutant AML, aggressive biology and resistance to conventional therapies are common [21, 31]. Both FLT3-ITD and Ras mutations were well represented in the sensitive cohort (Fig. 2A). MDM2 targeting could provide therapeutic benefit in these high-risk groups by restoring p53 function and potentially overcoming resistance mechanisms downstream of oncogenic signaling.

The efficacy of MDM2 degraders or inhibitors often relies on the functional presence of the p53 protein. In TP53 mutant or deleted leukemias, where p53 is absent or dysfunctional, MDM2 degraders are unlikely to be effective because there is no functional p53 to stabilize and activate. In our cohort, reduced activity of MD-265 was associated with mutant or deleted TP53 (Table 2). Additionally, subtypes with low expression of MDM2 or its homolog MDM4 showed resistance to MDM2-targeting therapies (Table 2 and Supplementary Fig 2). When MDM2 and MDM4 are not expressed, p53 regulation might be controlled by alternative mechanisms, or leukemic cells may rely on p53-independent programs that confer growth and survival advantages—possibilities that merit further exploration in MDM2 inhibitor/degrader-resistant disease.

In a humanized PDX model of AML, MD-265 provided a significant survival advantage (Fig. 4). Treatment reduced tumor burden and extended overall survival relative to both vehicle and an MDM2 inhibitor, with an acceptable tolerability profile in the tested schedules. In summary, we have shown a PROTAC MDM2 degrader that is efficacious in pre-clinical models at much lower doses, removes MDM2 protein effectively, and results in prolonged p53 inactivation. These findings support the translational potential of MDM2 degradation as a therapeutic modality. The only other MDM2 degrader to enter clinical trials is KT-253 (Kymera Therapeutics, NCT05775406) [32], and there is a need to explore and develop this class of therapeutics. A first-in-class oral MDM2 degrader is also in pre-clinical development [33]. Nevertheless, further preclinical and clinical studies are warranted to define specificity, off-target effects, and the potential development of resistance for this new class of drugs. Comparing the long-term effects of MDM2 degradation versus inhibition, including the durability of response after treatment cessation and the impact on normal hematopoiesis, will be critical for determining the overall therapeutic potential.

## Supplementary information

Supplemental Figures

## Data Availability

All data generated or analyzed during this study are included in this published article and its supplementary information.
