# Supplementary material for: Activity of PROTAC MDM2 degrader in primary leukemia cells and PDX models
Source: Leukemia. 2026 Apr 15;40(5):918–24. doi: 10.1038/s41375-026-02957-8 (PMC13149300; doi:10.1038/s41375-026-02957-8)
Supplement: Supplementary file 1 — Supplemental Figures [file 41375_2026_2957_MOESM1_ESM.pdf]

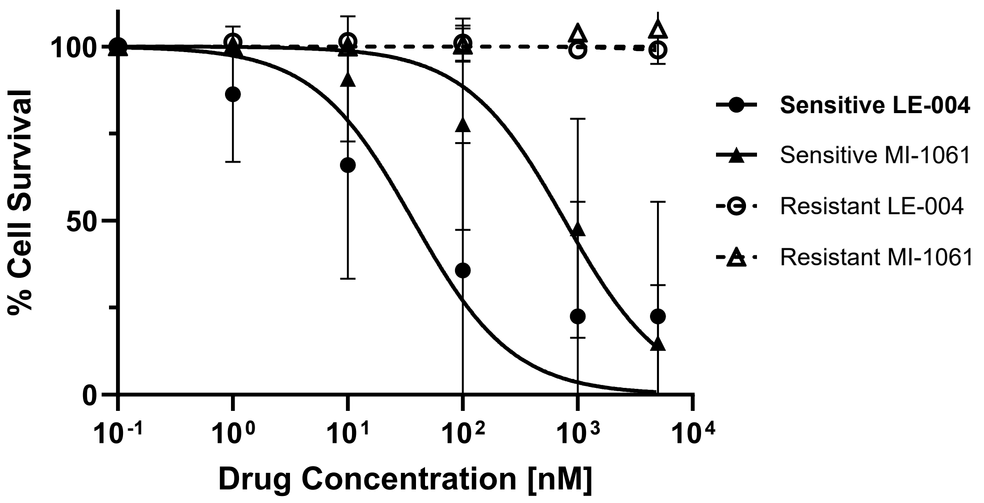

Supplementary Fig 1. Survival dose-response to MDM2 degraders LE-004 and the MDM2 inhibitor MI-1061 (N=17). Calculated IC50 values are shown in table.

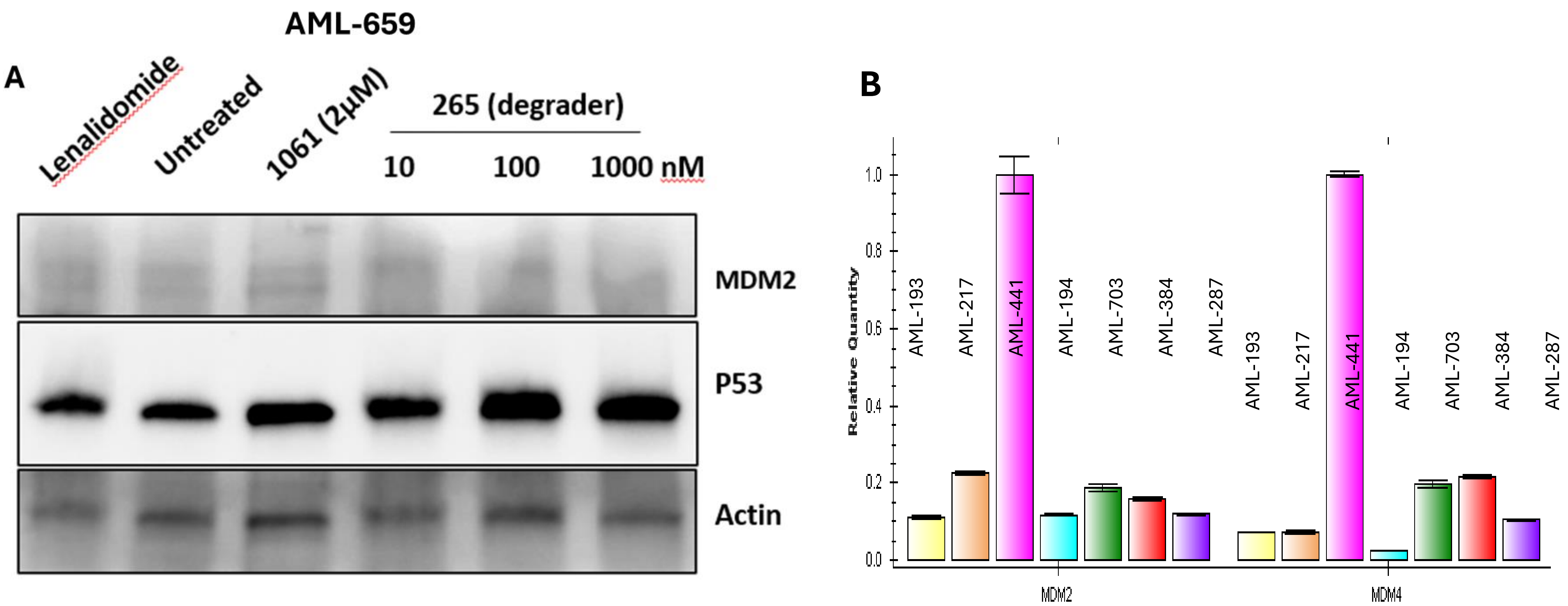

Supplementary Fig 2. Lack of MDM2 expression in AML LSCs resistant to MDM2 degrader. **A.** Immunoblot for MDM2, p53 and actin as loading control. **B.** Realtime quantitative PCR for MDM2 and MDM4.

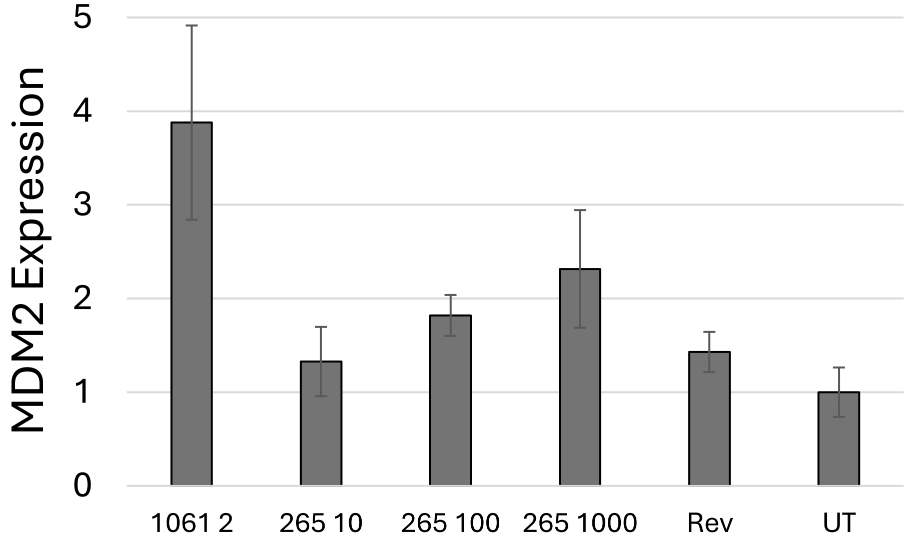

Supplementary Fig 3. MDM2 expression measured by real-time quantitative PCR, after treatment with MD-265 (10, 100, 1000nM), MI-1061(2 uM), and Revlimid.

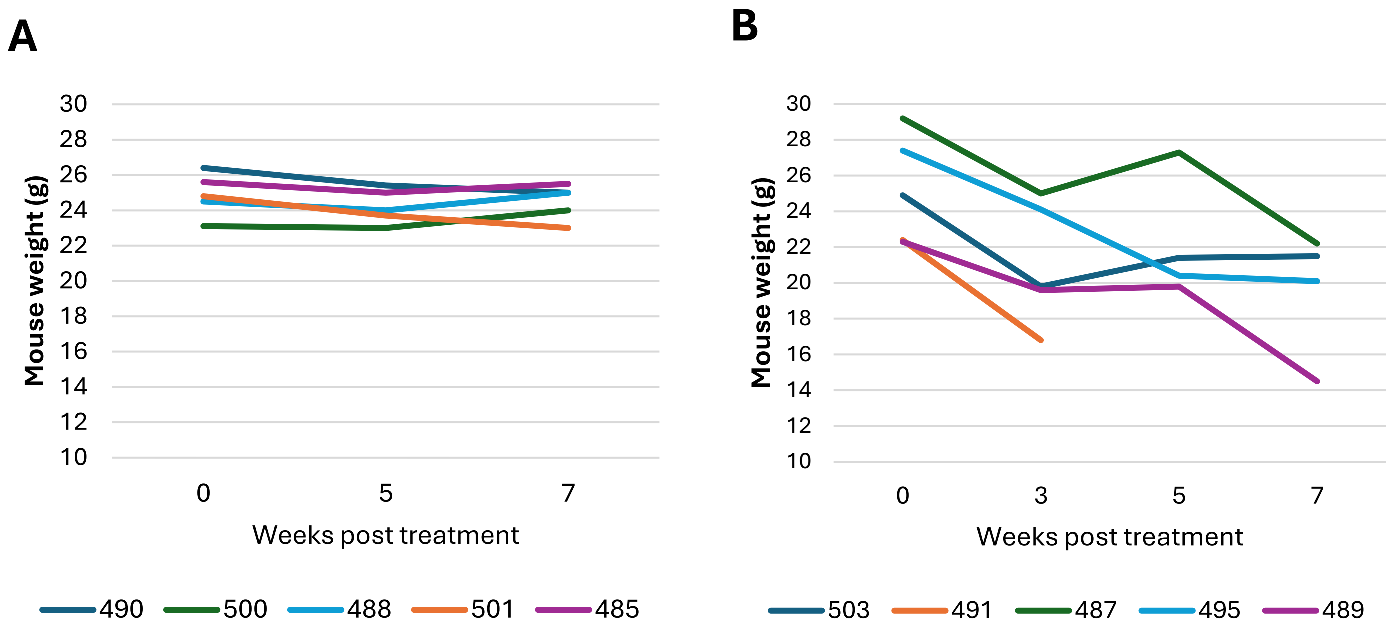

Supplementary Fig 4. Weight of mice after treatment with MD-265 (**panel A**) and MI-1061 (**panel B**). The lines indicate individual mice.
